# Prevalence of axial spondyloarthritis in Colombia: data from the National Health Registry 2017–2021

**DOI:** 10.1007/s10067-023-06799-y

**Published:** 2023-11-13

**Authors:** Julián E. Barahona-Correa, Nancy M. Herrera-Leaño, Santiago Bernal-Macías, Daniel G. Fernández-Ávila

**Affiliations:** 1 Department of Internal Medicine, Hospital Universitario San Ignacio, Bogota, Colombia; 2 Division of Rheumatology, Hospital Universitario San Ignacio, Bogota, Colombia; 3 School of Medicine, Pontificia Universidad Javeriana, Bogota, Colombia

**Keywords:** Axial spondyloarthritis, Epidemiology, Latin America, Registries, Spondyloarthritis

## Abstract

**Introduction:**

Registries allow ascertaining the epidemiology of chronic diseases such as axial spondyloarthritis (axSpA). The Colombian Ministry of Health has implemented a National Health Registry (SISPRO) that collects data from each medical contact in the system, which provides close to universal coverage (around 98%).

**Objective:**

To establish the 5-year prevalence of axSpA in Colombia, and to describe its demographics, using data from January 1st, 2017, to December 31st, 2021.

**Methods:**

We performed an observational, cross-sectional study using the International Statistical Classification of Diseases and Related Health Problems as search terms related to ax-SpA, based on SISPRO data. We estimated the prevalence using three approaches: (1) ankylosing spondylitis (AS) diagnoses; (2) diagnoses compatible with axSpA; and (3) diagnoses compatible with axSpA, including sacroiliitis. We calculated prevalence per 100,000 inhabitants.

**Results:**

Based on our three approaches, patients with a primary diagnosis compatible with ax-SpA ranged between 12,684 and 117,648, with an estimated 5-year adjusted prevalence between 26.3 and 244 cases per 100,000 inhabitants (0.03–0.2%). The male-to-female ratio ranged between 1.2:1 and 0.4:1, which was markedly skewed towards a higher prevalence in women when we included the code for sacroiliitis. We found the highest frequency of cases in the 50–54 years group. A differential prevalence was observed between different regions in our country, particularly in regions known to have European ancestors.

**Conclusion:**

This is the first study that describes demographic characteristics of ax-SpA in Colombia and offers valuable information for stakeholders.

**Table:** 

**Key Points** *• Using the official country-level health database, the prevalence of axSpA in Colombia ranges between 26.3 and 244 cases per 100,000 inhabitants (0.03% - 0.2%)* *• The prevalence of axSpA peaked among the 50-54 years patient group, suggesting an increased survival* *• Nations with a substantial admixture, such as Colombia, may present a differential prevalence of axSpA among regions within the country* *• Including the ICD-10 code for sacroiliitis (M46.1) in epidemiological studies probably overestimates the frequency of axSpA*

**Supplementary Information:**

The online version contains supplementary material available at 10.1007/s10067-023-06799-y.

## Introduction

The continuum of axial spondyloarthritis (axSpA) is an expanding field in rheumatology practice. The emerging terminology classifies axSpA in radiographic (r-axSpA) and non-radiographic (r-axSpA) [1, 2]. AxSpA presents a high societal burden predominantly affecting patients during their most productive years, usually younger than 45 years old, who may present a higher risk of comorbidities [1, 3, 4]. In addition, a significant diagnostic delay is usually observed and may be as long as 14 years [5]. Prevalence studies provide stakeholders with information on the burden of a condition, its distribution, and demographic characteristics, thus, allowing the development of health policies and resource allocation [6].

A systematic review and meta-regression analysis suggested a global prevalence of SpA (including every subtype) ranging between 0.2 and 1.6%. Regarding axSpA, the authors reported a prevalence between 0.5 and 0.7% [7]. The estimated prevalence of AS in Latin America was 0.14% (0.02–0.34) [7]. Recently, Citera et al. explored the prevalence of SpA in Latin America. Regarding axSpA, the prevalence ranged between 0.2 and 0.9%, whereas AS ranged between 0.02 and 0.8% [8]. Using an in-field epidemiologic strategy, a local study in Colombia reported a prevalence of AS of 0.11% (0.03–0.36) [9].

Based on data provided by the official administrative registry of the Colombian Ministry of Health, the present study aimed to establish the prevalence of axSpA in Colombia and to describe its demographics.

## Methods

Colombia has one of the broadest health coverages in Latin America: 98.54% of the 51.6 million inhabitants as of August 2022, according to official data from the Colombian Ministry of Health [10]. The Ministry developed an information database called SISPRO, which stores and processes the essential data the system requires for its regulation and control processes. Demographics and clinical data are collected by medical staff during each outpatient or inpatient medical contact and are grouped in the Individual Health Services Delivery Registry (Registro Individual de Prestación de Servicios de Salud, RIPS, by its Spanish acronym). These databases are publicly available for scientific analysis (http://www.sispro.gov.co/); we retrieved the information to carry out this study from the online dynamic tables. Currently, the database holds information up to 2021. We obtained the information for the whole country between January 1st, 2017, and December 31st, 2021. We analyzed the RIPS database using the International Statistical Classification of Diseases and Related Health Problems 10th Revision (ICD-10) codes for conditions compatible with axSpA (Supplementary Table 1). In order to offer unbiased data, we estimated the prevalence using three approaches: (1) AS diagnoses (M081 and M45X); (2) diagnoses compatible with axSpA (M081, M45X, M46.0, M46.8, M46.9); (3) diagnoses compatible with axSpA, including sacroiliitis (M46.1). Diagnostic criteria for axSpA in the SISPRO database are not standardized and depend on each physician. In addition, only the “main diagnosis” is included, potentially leading to underreporting in patients with multiple diagnoses. However, we could assume axSpA should classify as the primary diagnosis in most, if not all, cases. Following the Strengthening the Reporting of Observational Studies in Epidemiology (STROBE) guidelines, we analyzed the distribution in 5-year age groups according to data from the most recent census (2018) [11]. We calculated prevalence per 100,000 inhabitants using as a numerator the number of patients diagnosed with a code compatible with axSpA (counted once). The denominator was the number of inhabitants reported by the National Administrative Department of Statistics (Departamento Administrativo Nacional de Estadística, DANE, by its Spanish acronym) in each age group or geographical area. The prevalence was adjusted by age, gender, and region using the direct method. Data were recorded and analyzed using Microsoft Excel (Microsoft Corp., Redmond, WA, USA).

## Results

### AS diagnoses (M081 and M45X)

**Fig. 1:**
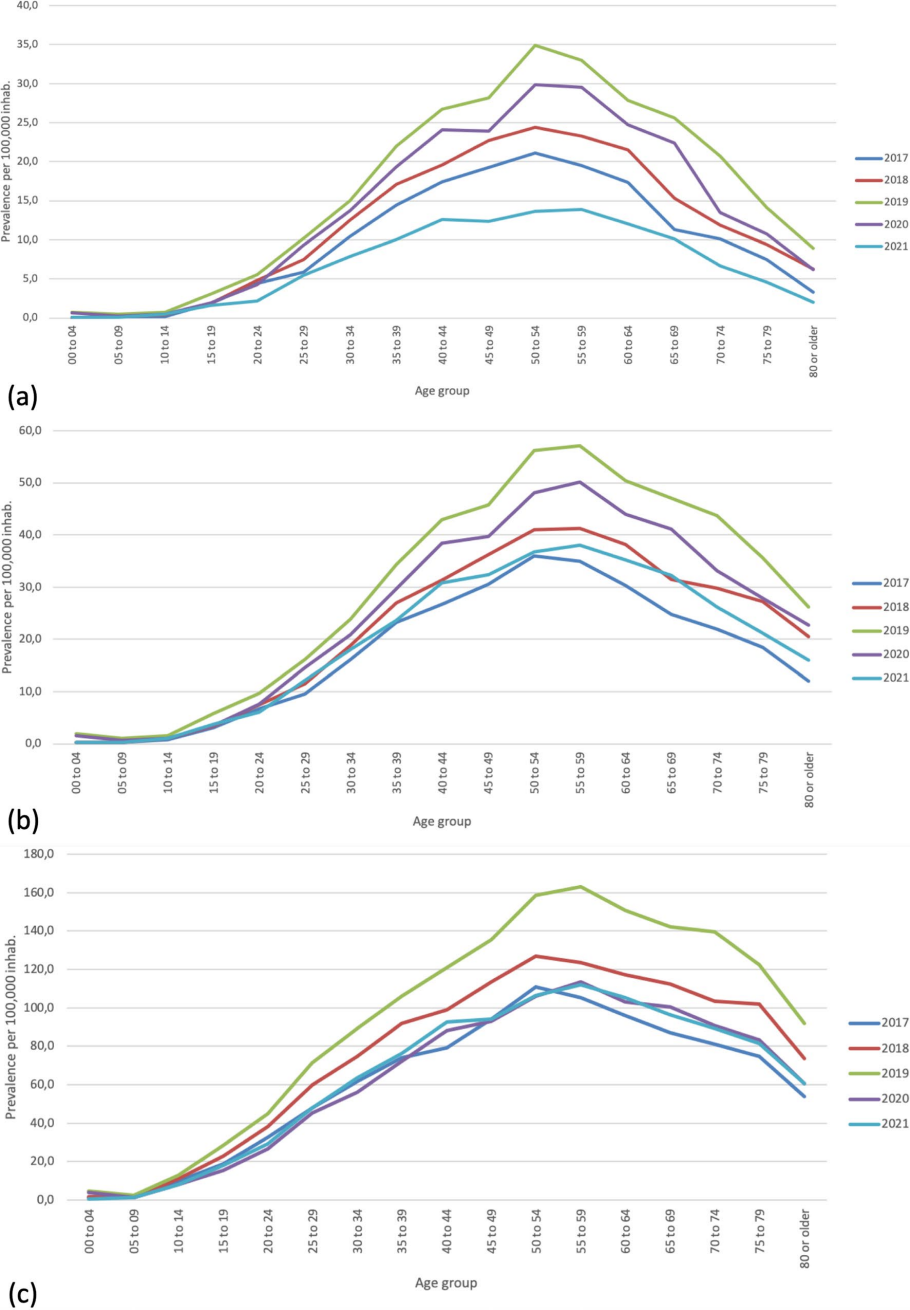
Age-specific prevalence of **a** patients with ankylosing spondylitis; **b** patients with diagnoses compatible with axSpA; and **c** patients with diagnoses compatible with axSpA, including sacroiliitis during the years 2017–2021. Prevalence calculated with the average population of the period as denominator per 100,000 population

**Fig. 2:**
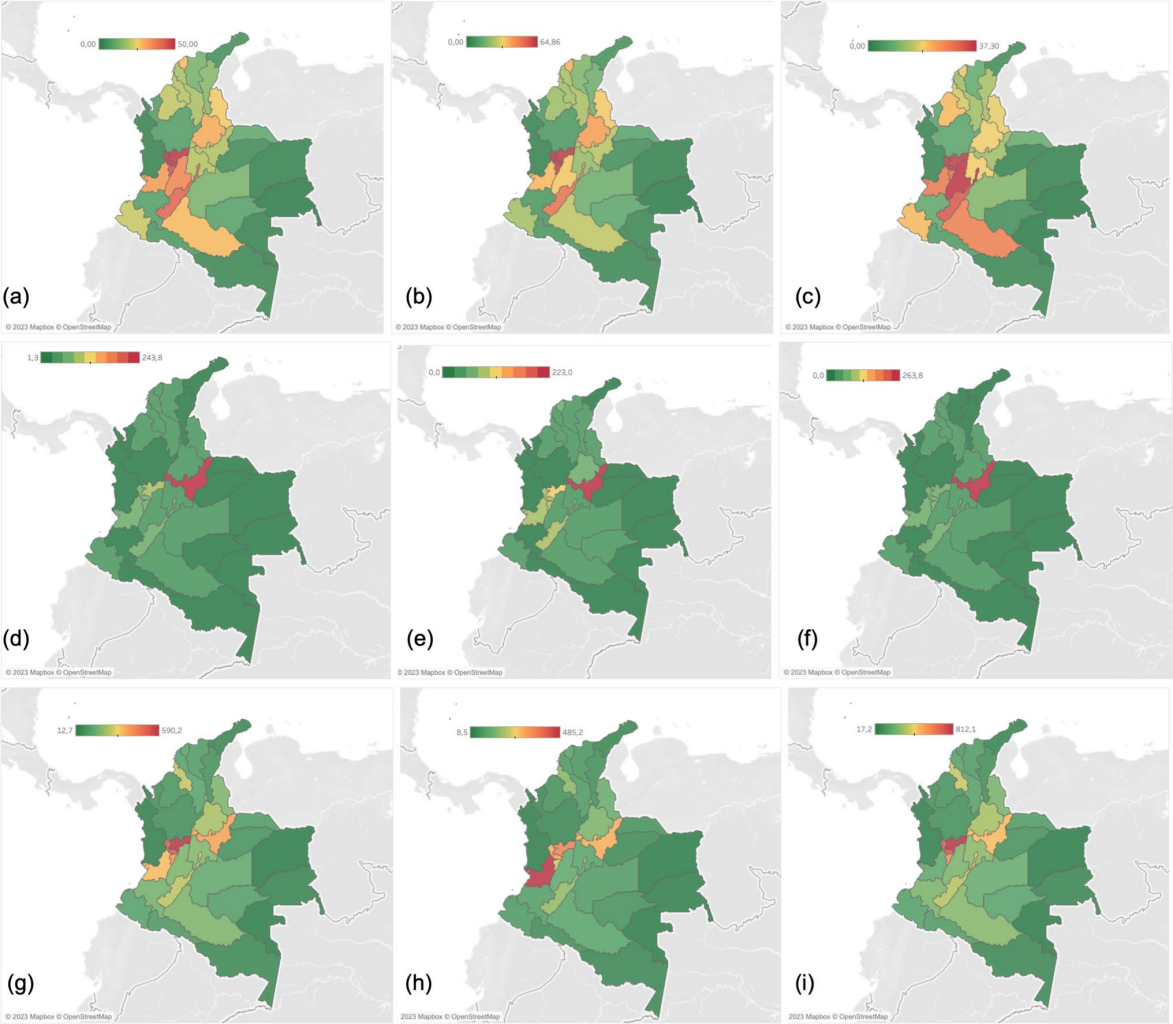
Geographic distribution of the adjusted prevalence of patients with ankylosing spondylitis [M081, M45X; **a** global, **b** male, and **c** female], patients with diagnoses compatible with axSpA [M081, M45X, M46.0, M46.8, M47.9; **d** global, **e** male, and **f** female], and patients with diagnoses compatible with axSpA, including sacroiliitis [M081, M45X, M46.0, M46.1, M46.8, M47.9; **g** global, **h** male, and **i** female] for the period by departments and by gender. Prevalence is calculated with the average population of the period as denominator per 100,000 population

We identified 12,684 individual patients (“cases”) with a primary diagnosis of AS during the five years. To calculate prevalence, we used the estimated population of the 2018 census as a denominator: 48,258,494 inhabitants. Thus, we estimated a 5-year adjusted prevalence of 26.3 cases per 100,000 inhabitants (0.03%). The male and female prevalence was 29.2 and 23.5 cases per 100,000 inhabitants, respectively, with a male-to-female ratio of 1.2:1. Concerning age group prevalence, we observed a progressive increase reaching its highest prevalence in the 50–54 years group (Fig. 1a, Supplementary material 2). Table 1 presents prevalence by age group and gender, and Fig. 2 depicts the geographic distribution by the department. The general prevalence ranged between 0 and 50 cases per 100,000 inhabitants; male prevalence ranged between 0 and 64.9 cases, whereas female prevalence ranged between 0 and 37.3 cases. The highest general prevalence was present in Risaralda, Caldas, and Quindio. In the case of men, Bogotá, Colombia’s capital city, showed the highest prevalence. However, we detected the highest prevalence in women in Bogotá, Quindio, and Tolima.

**Table 1:** Patients with a main diagnosis of ankylosing spondylitis (M081, M45X) by gender and age group between 2017 and 2021

Age group (years)	Male		Female		Total population
	Patients	Prevalence^a^	Patients	Prevalence^a^	
0–04	32	1.6	27	1.5	60
05–09	20	1	22	1.2	42
10–14	49	2.4	24	1.2	73
15–19	205	9.7	157	7.7	363
20–24	364	17.2	212	10.1	576
25–29	568	28.8	380	19	948
30–34	736	41.2	592	31.9	1328
35–39	929	56.2	772	43.8	1701
40–44	983	68.6	850	54	1833
45–49	1001	74.6	839	55.5	1840
50–54	1090	84.5	939	63.5	2032
55–59	989	87.4	825	62.8	1814
60–64	725	80	551	51.8	1276
65–69	480	69.8	331	40.7	811
70–74	273	55.4	198	33.4	471
75–79	145	44.6	108	27.1	253
80 or older	116	28.3	85	16.1	201
Total	6887	29.2	5792	23.5	12,684

^**a**^Calculated with the average population of the period as denominator per 100,000 population. Gender information was missing for 5 patients

### Diagnoses compatible with axSpA (M081, M45X, M46.0, M46.8, M47.9)

We identified 26,488 patients (“cases”) with a diagnosis compatible with axSpA during the five years. We estimated a 5-year adjusted prevalence of 55 cases per 100,000 inhabitants (0.05 %). The male and female prevalence was 52 and 57 cases per 100,000 inhabitants, respectively, with a male-to-female ratio of 0.9:1. Concerning age group prevalence, we observed a progressive increase reaching its highest prevalence in the 50–54 years group (Fig. 1b, Supplementary material 3). Table 2 presents prevalence by age group and gender. Figure 2 depicts the geographic distribution by the department. The general prevalence ranged between 1.3 and 337.3 cases per 100,000 inhabitants; male prevalence ranged between 0 and 243.8 cases, whereas female prevalence ranged between 0 and 426.4 cases. The highest general prevalence was in San Andrés Island, Boyacá, and Caldas. We observed a similar distribution for both men and women.

**Table 2:** Patients with diagnoses compatible with axSpA (M081, M45X, M46.0, M46.8, M46.9) by gender and age group between 2017 and 2021

Age group (years)	Male		Female		Total population
	Patients	Prevalence^a^	Patients	Prevalence^a^	
0–04	84	4	72	4	158
05–09	42	2	48	3	90
10–14	105	5	83	4	188
15–19	355	16	318	16	676
20–24	590	25	477	23	1067
25–29	864	42	784	39	1648
30–34	1113	62	1154	62	2267
35–39	1413	88	1575	89	2988
40–44	1559	109	1717	109	3276
45–49	1601	121	1838	122	3439
50–54	1753	142	2173	147	3929
55–59	1646	152	2062	157	3708
60–64	1298	141	1470	138	2768
65–69	973	131	987	121	1960
70–74	609	123	722	122	1331
75–79	383	111	421	106	804
80 or older	391	85	406	77	797
Total	12,303	52	14,177	57	26,488

^**a**^Calculated with the average population of the period as denominator per 100,000 population. Gender information was missing for 8 patients

### Diagnoses compatible with axSpA, including sacroiliitis (M081, M45X, M46.0, M46.8, M47.9, M46.1)

During the 5 years, we identified 117,648 individual patients (“cases”) with a diagnosis compatible with axSpA, including sacroiliitis. We estimated a 5-year adjusted prevalence of 244 cases per 100,000 inhabitants (0.24%). The male and female prevalence was 139 and 344 cases per 100,000 inhabitants, respectively, with a male-to-female ratio of 0.4:1. Concerning age group prevalence, we observed a progressive increase reaching its highest prevalence in the 50–54 years group (Fig. 1c, Supplementary material 4). Noteworthy, we detected a high frequency of cases in patients younger than 18. Table 3 presents prevalence by age group and gender, and Fig. 2 depicts the geographic distribution by the department. The general prevalence ranged between 12.7 and 590.2 cases per 100,000 inhabitants; male prevalence ranged between 8.5 and 485.2 cases, whereas female prevalence ranged between 17.2 and 812.1 cases.

**Table 3:** Patients with diagnoses compatible with axSpA, including sacroiliitis (M081, M45X, M46.0, M46.1, M46.8, M46.9) by gender and age group between 2017 and 2021

Age group (years)	Male		Female		Total population
	Patients	Prevalence^a^	Patients	Prevalence^a^	
0–04	192	10	255	14	449
05–09	125	6	165	9	291
10–14	514	25	1383	72	1897
15–19	1187	56	2864	141	4061
20–24	1731	82	4753	227	6488
25–29	2570	131	6939	347	9510
30–34	3045	170	7689	414	10,734
35–39	3526	213	8471	481	12,000
40–44	3430	239	8512	541	11,942
45–49	3453	257	9154	606	12,607
50–54	3612	280	10,400	704	14,015
55–59	3486	308	9315	709	12,801
60–64	2849	314	6915	650	9765
65–69	2247	327	4896	602	7143
70–74	1607	326	3335	563	4942
75–79	1030	317	2069	520	3099
80 or older	1032	252	1960	371	2992
Total	32,740	139	84,883	344	117,648

^**a**^Calculated with the average population of the period as denominator per 100,000 population. Gender information was missing for 25 patients

The highest general prevalence was in Caldas, San Andrés Island, and Risaralda. We observed a similar prevalence distribution for men and women, although a high prevalence was observed Valle del cauca for men.

## Discussion

Using the National Health Registry data, we estimated the prevalence of axSpA in Colombia using three different data-retrieving approaches. Our data suggest that the prevalence lies between 26.3 and 244 cases per 100,000 inhabitants (0.03–0.2%). The male-to-female ratio was different between the three approaches, with a higher proportion of males in the AS-only analysis, almost equal in the axSpA-compatible codes analysis, and a higher proportion of females when we included sacroiliitis. To the best of our knowledge, our report is the first to assess the prevalence of axSpA in Colombia using the official country-level health database.

Previous reports have used ICD codes to explore epidemiological data on axSpA [12–15]. Due to the potential heterogeneity of ICD codes regarding axSpA, we applied three search strategies. We found a pooled 5-year unadjusted prevalence of axSpA (including sacroiliitis) within the range of that reported previously (0.2% and 0.9%) [7, 8]. However, after reviewing the demographics of including the code for sacroiliitis (M46.1, Table 3), the male-to-female distribution ratio was markedly skewed towards a higher prevalence in women. Although the prevalence of axSpA (particularly AS) has presented a steep increase during the last decade, the prevalence is still higher among male patients [16, 17]. Our findings appear to be mainly driven by the inclusion of the “sacroiliitis” code. Sacroiliitis presents a myriad of causes found in the general population, including different strains (e.g., sports, pregnancy) [3, 18]. As axial involvement in female patients with axSpA is less frequent [19], this suggests other causes for sacroiliitis. Based on this rationale, the most reliable prevalence estimate of axSpA is our second approach, which excludes sacroiliitis. Thus, we estimated a 5-year adjusted prevalence of 56 cases per 100,000 inhabitants (0.06 %) with a male-to-female ratio of 0.9:1, comparable to previously reported global and regional estimates [7, 8].

Regarding AS prevalence, our estimate (27.1 per 100,000 inhabitants), was significantly lower than those reported in a systematic review, in which Dean et al. found over 100 cases per 100,000 inhabitants in Asia, Europe, and North and Latin America; only one study from Cuba represented Latin population [20]. Another systematic review published in 2016 reported a prevalence of 0.14% (0.02–0.34 %) in Latin America [7]. Our findings lie within the confidence interval of this report [7]. Further, a systematic review focused on Latin America was published in 2021 and reported an AS prevalence of 0.02–0.8% [8]. Finally, Londoño et al. explored the prevalence of rheumatic diseases in Colombia using an in-field strategy and found a similar prevalence of AS [9]. Regarding age distribution, we found that prevalence peaked among the 50–54 years patient group, probably due to increased survival [16]. Figure 1 showed a higher prevalence in 2019 when compared to other periods. We hypothesize that a more stringent data capture was implemented within the healthcare system, which allowed a better data quality. This is illustrated by the increasing prevalence between 2017 and 2018 that peaked in 2019. However, as this database relies on in-person medical encounters, a gradual decrease is observed in 2020 and 2021 probably associated to COVID-19 pandemic.

The existence of regional differences in the prevalence of axSpA may be partly explained by the prevalence of HLA-B27 [7]. This genotype is frequently found in people with European ancestry [21]. Due to the arrival of people of European and African ancestry in Colombia in the 15th century, a process of racial mixing with the native population occurred. The largest number of people with European ancestors are found in the Andean, Caribbean, and Orinoquia regions [22, 23]. Accordingly, we recorded the highest general prevalence of AS in this region. Based on these facts, two approaches may be explored. On the one hand, the prevalence of HLA-B27 in the general population suggests a prevalence between 0 and 4.5%, particularly in the Andean region [24–33]. Noteworthy, we observed a high prevalence in the Archipelago of San Andres, an isolated landmass found in the Caribbean sea close to countries such as Jamaica, whose population is a product of a substantial admixture between enslaved African, European colonizers, and Amerindian groups [34]. Although the HLA epidemiology from the Archipelago is unknown, a study in the Jamaican black population did not find B27 antigen among participants [35]. SpA in African patients is currently considered rare, partly due to a low prevalence of HLA-B27. [36]. Thus, the observed high prevalence may be due to the presence of European genes, although more evidence is warranted.

On the other hand, the prevalence of HLA-B27 in Colombian patients with axSpA (mainly AS) ranges between 25 and 90%; these data were mainly obtained from patients from the Andean region [37–41]. Further, the frequency of HLA-B27 and HLA-B15 in patients with axSpA or AS is higher than that in healthy controls [41]. The HLA-B15 family is widely heterogenous and includes B15, B-46, B-62, B-63, B-75, B-76, and B-77 antigens [42]. Previous studies in Colombia have described a prevalence of HLA-B15 between 2 and 6% in healthy people from different regions, but as high as 26% in indigenous people [26, 28, 32, 35, 43]. The prevalence in Colombian patients with SpA lies between 3 and 20% [40, 41, 44]. Interestingly, the presence of HLA-B15 in patients with SpA was associated with peripheral disease, whereas HLA-B27 was associated with axial manifestations [44]. To date, it is considered that HLA-B27 explains only 13 to 50% of the presence of AS [33].

We acknowledge some drawbacks in our study. First, as the retrieved data come from physicians coding in the medical record, the compliance of diagnosis with the classification criteria is unknown; the prevalence may be under or overestimated. Nonetheless, this database comprises the main statistics from the Colombian Ministry of Health and provides the foundation for public health policy decisions. Second, the prevalence may be underestimated in underserved areas, as the formal diagnosis may be registered in larger cities due to the concentration of rheumatologists. Third, comparing our data with the prevalence reported in previous studies is challenging, as different methodologies have been used.

## Conclusion

Based on the National Health Registry data, we estimated a prevalence of axSpA in Colombia between 26.3 and 244 cases per 100,000 inhabitants (0.03–0.2%). A differential prevalence was observed between different regions in our country. From an individual and societal perspective, axSpA is a burdensome disease. Thus, epidemiological data will allow stakeholders to allocate resources to improve diagnosis and treatment strategies.

### Supplementary information

ESM 1

## Data Availability

All data will be available at request to the authors.
