# Supplementary material for: Prevalence of axial spondyloarthritis in Colombia: data from the National Health Registry 2017–2021
Source: Clin Rheumatol. 2023 Nov 13;43(1):49–57. doi: 10.1007/s10067-023-06799-y (PMC10774146; doi:10.1007/s10067-023-06799-y)
Supplement: Supplementary file 1 — ESM 1 [file 10067_2023_6799_MOESM1_ESM.docx]

Supplementary Material

S1. Included codes from the International Statistical Classification of Diseases and Related Health Problems 10th Revision

| M081 Juvenile ankylosing spondylitis |
| --- |
| M45X Ankylosing spondylitis |
| M46.0 Spinal enthesopathy |
| M46.8 Other specified inflammatory spondylopathies |
| M46.9 Inflammatory spondylopathy, unspecified |
| M46.1 Sacroiliitis, not elsewhere classified |

S2. Patients with a main diagnosis of ankylosing spondylitis (M081, M45X) according to age group between 2017 and 2021

| **Age group (years)** | **2017** | **2018** | **2019** | **2020** | **2021** | **Total patients^a^** |
| --- | --- | --- | --- | --- | --- | --- |
| **0 - 04** | 1 | 2 | 28 | 25 | 4 | 60 |
| **05 - 09** | 6 | 6 | 20 | 10 | 5 | 42 |
| **10 - 14** | 7 | 15 | 28 | 19 | 20 | 73 |
| **15 - 19** | 78 | 78 | 127 | 79 | 66 | 363 |
| **20 - 24** | 185 | 202 | 233 | 181 | 92 | 576 |
| **25 - 29** | 232 | 298 | 406 | 369 | 217 | 948 |
| **30 - 34** | 381 | 455 | 546 | 500 | 287 | 1,328 |
| **35 - 39** | 493 | 585 | 750 | 661 | 343 | 1,701 |
| **40 - 44** | 524 | 589 | 803 | 724 | 379 | 1,833 |
| **45 - 49** | 550 | 647 | 804 | 683 | 353 | 1,840 |
| **50 - 54** | 584 | 676 | 966 | 827 | 377 | 2,032 |
| **55 - 59** | 478 | 570 | 806 | 722 | 340 | 1,814 |
| **60 - 64** | 342 | 423 | 548 | 487 | 237 | 1,276 |
| **65 - 69** | 170 | 230 | 384 | 336 | 152 | 811 |
| **70 - 74** | 110 | 129 | 225 | 146 | 72 | 471 |
| **75 - 79** | 54 | 68 | 102 | 78 | 33 | 253 |
| **80 or older** | 31 | 59 | 84 | 58 | 19 | 201 |
| **Total** | 3,986 | 4,722 | 6,427 | 5,520 | 2,864 | 12,684 |
| ^a^ Number of people who were attended at some point during the 5-year period.  ^b^ Calculated with the average population of the period as denominator per 100,000 population. | | | | | | |

S3. Patients with diagnoses compatible with axSpA (M081, M45X, M46.0, M46.8, M46.9) according to age group between 2017 and 2021

| **Age group (years)** | **2017** | **2018** | **2019** | **2020** | **2021** | **Total patients^a^** |
| --- | --- | --- | --- | --- | --- | --- |
| **0 - 04** | 9 | 9 | 73 | 59 | 8 | 158 |
| **05 - 09** | 10 | 9 | 42 | 25 | 10 | 90 |
| **10 - 14** | 31 | 43 | 60 | 43 | 41 | 188 |
| **15 - 19** | 131 | 140 | 242 | 146 | 154 | 676 |
| **20 - 24** | 285 | 316 | 405 | 322 | 253 | 1,067 |
| **25 - 29** | 380 | 458 | 640 | 579 | 481 | 1,648 |
| **30 - 34** | 588 | 687 | 871 | 760 | 656 | 2,267 |
| **35 - 39** | 794 | 920 | 1,170 | 1,012 | 806 | 2,988 |
| **40 - 44** | 803 | 942 | 1,291 | 1,154 | 926 | 3,276 |
| **45 - 49** | 871 | 1,035 | 1,306 | 1,133 | 923 | 3,439 |
| **50 - 54** | 997 | 1,136 | 1,557 | 1,331 | 1,019 | 3,929 |
| **55 - 59** | 854 | 1,009 | 1,397 | 1,228 | 932 | 3,708 |
| **60 - 64** | 598 | 751 | 994 | 867 | 694 | 2,768 |
| **65 - 69** | 372 | 473 | 706 | 617 | 484 | 1,960 |
| **70 - 74** | 238 | 323 | 475 | 360 | 284 | 1,331 |
| **75 - 79** | 133 | 197 | 257 | 201 | 153 | 804 |
| **80 or older** | 113 | 192 | 246 | 213 | 150 | 797 |
| **Total** | 6,857 | 8,173 | 11,083 | 9,449 | 7,590 | 26,488 |
| ^a^ Number of people who were attended at some point during the 5-year period.  ^b^ Calculated with the average population of the period as denominator per 100,000 population. | | | | | | |

S4. Patients with diagnoses compatible with axSpA, including sacroiliitis (M081, M45X, M46.0, M46.1, M46.8, M46.9) according to age group between 2017 and 2021

| **Age group (years)** | **2017** | **2018** | **2019** | **2020** | **2021** | **Total patients^a^** |
| --- | --- | --- | --- | --- | --- | --- |
| **0 - 04** | 53 | 59 | 173 | 144 | 22 | 449 |
| **05 - 09** | 49 | 51 | 95 | 51 | 52 | 291 |
| **10 - 14** | 389 | 443 | 509 | 311 | 315 | 1,897 |
| **15 - 19** | 774 | 949 | 1,184 | 647 | 756 | 4,061 |
| **20 - 24** | 1,376 | 1,605 | 1,886 | 1,125 | 1,234 | 6,488 |
| **25 - 29** | 1,905 | 2,373 | 2,828 | 1,803 | 1,904 | 9,510 |
| **30 - 34** | 2,251 | 2,728 | 3,259 | 2,045 | 2,322 | 10,734 |
| **35 - 39** | 2,524 | 3,142 | 3,620 | 2,469 | 2,598 | 12,000 |
| **40 - 44** | 2,387 | 2,979 | 3,642 | 2,651 | 2,791 | 11,942 |
| **45 - 49** | 2,688 | 3,234 | 3,864 | 2,655 | 2,684 | 12,607 |
| **50 - 54** | 3,071 | 3,510 | 4,396 | 2,936 | 2,947 | 14,015 |
| **55 - 59** | 2,572 | 3,026 | 3,992 | 2,777 | 2,743 | 12,801 |
| **60 - 64** | 1,894 | 2,306 | 2,967 | 2,029 | 2,072 | 9,765 |
| **65 - 69** | 1,309 | 1,689 | 2,135 | 1,507 | 1,447 | 7,143 |
| **70 - 74** | 881 | 1,121 | 1,516 | 983 | 970 | 4,942 |
| **75 - 79** | 541 | 736 | 886 | 603 | 588 | 3,099 |
| **80 or older** | 505 | 690 | 862 | 569 | 567 | 2,992 |
| **Total** | 24,642 | 29,900 | 36,832 | 24,440 | 25,412 | 117,648 |
| ^a^ Number of people who were attended at some point during the 5-year period.  ^b^ Calculated with the average population of the period as denominator per 100,000 population. | | | | | | |
